# Occupational Exposure to Benzene and Chromosomal Structural Aberrations in the Sperm of Chinese Men

**DOI:** 10.1289/ehp.1103921

**Published:** 2011-11-15

**Authors:** Francesco Marchetti, Brenda Eskenazi, Rosana H. Weldon, Guilan Li, Luoping Zhang, Stephen M. Rappaport, Thomas E. Schmid, Caihong Xing, Elaine Kurtovich, Andrew J. Wyrobek

**Affiliations:** 1 Life Sciences Division, Lawrence Berkeley National Laboratory, Berkeley, California, USA; 2 Center for Environmental Research and Children’s Health, School of Public Health, University of California, Berkeley, California, USA; 3 National Institute of Occupational Health and Poison Control, Chinese Center for Disease Control and Prevention, Beijing, China; 4 Center for Exposure Biology, School of Public Health, University of California, Berkeley, California, USA

**Keywords:** benzene, chromosome 1, germ cells, sperm fluorescence *in situ* hybridization, structural aberrations

## Abstract

Background: Benzene is an industrial chemical that causes blood disorders, including acute myeloid leukemia. We previously reported that occupational exposures near the U.S. Occupational Safety and Health Administration permissible exposure limit (8 hr) of 1 ppm was associated with sperm aneuploidy.

Objective: We investigated whether occupational exposures near 1 ppm increase the incidence of sperm carrying structural chromosomal aberrations.

Methods: We applied a sperm fluorescence *in situ* hybridization assay to measure frequencies of sperm carrying partial chromosomal duplications or deletions of 1cen or 1p36.3 or breaks within 1cen-1q12 among 30 benzene-exposed and 11 unexposed workers in Tianjin, China, as part of the China Benzene and Sperm Study (C-BASS). Exposed workers were categorized into low-, moderate-, and high-exposure groups based on urinary benzene (medians: 2.9, 11.0, and 110.6 µg/L, respectively). Median air benzene concentrations in the three exposure groups were 1.2, 3.7, and 8.4 ppm, respectively.

Results: Adjusted incidence rate ratios (IRRs) and 95% confidence intervals (CIs) for all structural aberrations combined were 1.42 (95% CI: 1.10, 1.83), 1.44 (95% CI: 1.12, 1.85), and 1.75 (95% CI: 1.36, 2.24) and for deletion of 1p36.3 alone were 4.31 (95% CI: 1.18, 15.78), 6.02 (95% CI: 1.69, 21.39), and 7.88 (95% CI: 2.21, 28.05) for men with low, moderate, and high exposure, respectively, compared with unexposed men. Chromosome breaks were significantly increased in the high-exposure group [IRR 1.49 (95% CI: 1.10, 2.02)].

Conclusions: Occupational exposures to benzene were associated with increased incidence of chromosomally defective sperm, raising concerns for worker infertility and spontaneous abortions as well as mental retardation and inherited defects in their children. Our sperm findings point to benzene as a possible risk factor for *de novo* 1p36 deletion syndrome. Because chromosomal aberrations in sperm can arise from defective stem cells/spermatogonia, our findings raise concerns that occupational exposure to benzene may have persistent reproductive effects in formerly exposed workers.

Benzene is an important industrial chemical with > 2 billion pounds produced every year in the United States. Low-level exposures (< 5 ppb) to benzene, widespread in the U.S. population, are primarily due to smoking, gasoline fumes, and vehicle emissions (Hricko 1994). Early epidemiological cohort studies found that benzene is associated with an increased risk of leukemia at high levels (around 10 ppm average or 40 ppm-years) (Hayes et al. 1997; Yin et al. 1996, 1987a, 1987b), whereas more recent studies found excess leukemia risk associated with levels of exposure as low as 0.8–1.6 ppm or 2–4 ppm-years of cumulative exposure (Glass et al. 2003, 2004; Hayes et al. 2001). Benzene is hematotoxic, and in a large study of more than 400 workers, almost all blood cell counts were significantly decreased, even in individuals exposed to < 1 ppm benzene [mean ± SD of exposed individuals 0.57 ± 0.24 ppm, and mean of unexposed individuals ≤ 0.04 ppm (Lan et al. 2004)]. Therefore, benzene is highly regulated, with the U.S. permissible exposure limit (PEL; 8-hr time-weighted average) set at 1 ppm by the Occupational Safety and Health Administration (OSHA 1987). Although significant international progress has been seen in reducing occupational exposure to benzene, workers in some countries still experience levels of benzene well above the U.S. PEL (Liang et al. 2005).

Specific chromosomal aneuploidies and aberrations implicated in leukemia have been detected in the blood cells of benzene-related leukemia patients as well as in healthy benzene-exposed workers, suggesting that these abnormalities precede and may be a potential mechanism underlying benzene-induced leukemia (Zhang et al. 2002, 2005, 2011). Recent findings from our group and others suggest that occupational exposure to benzene induces aneuploidies in sperm (Li et al. 2001; Liu S et al. 2000; Zhao et al. 2004), even in workers exposed at or below the U.S. PEL (Xing et al. 2010). In addition, in Chinese workers exposed to high doses of benzene (> 10 ppm), exposure appears to increase terminal duplications and deletions for chromosome 1 and increase centromeric duplications and deletions for chromosome 1 in sperm (Liu XX et al. 2003). No data are yet available on whether benzene exposures near the U.S. PEL have detrimental effects on chromosomal structural aberrations in sperm.

In humans, approximately 0.6% of newborns carry constitutive chromosomal abnormalities in the form of aneuploidies or structural aberrations (Jacobs et al. 1989; Shelby et al. 1993). Although the incidence of children born with chromosomal aberrations is lower than for aneuploidies, these types of aberrations have approximately 80% paternal contribution and result in severe health consequences (Chandley 1991; Crow 2001). Inherited chromosomal aberrations of paternal origin can arise in spermatogonia, meiotic cells, or postmeiotic cells by different mechanisms. Sperm carrying partial chromosomal duplications and deletions in sperm can arise from stem cells carrying reciprocal translocations (van Hummelen et al. 1997). In healthy men, sperm with chromosomal breaks are more prevalent than sperm with partial chromosomal duplications and deletions (Sloter et al. 2000), and both these classes of aberrations increase with age (Sloter et al. 2004). The postmeiotic period of spermatogenesis appears to be very sensitive to the induction of DNA lesions that can lead to DNA strand breaks directly in sperm or after fertilization (Marchetti and Wyrobek 2005; Olsen et al. 2005). Unrepaired or misrepaired DNA damage in sperm can result in chromosomally abnormal offspring with a variety of heritable diseases and dysmorphologies (Marchetti and Wyrobek 2005).

The objective of our study was to investigate the prevalence of sperm carrying structural chromosomal abnormalities in a group of Chinese workers exposed to levels of benzene near the U.S. PEL. We used the sperm alpha, classical, midi (ACM)-fluorescence *in situ* hybridization (FISH) assay that employs DNA probes specific for three regions of chromosome 1 (Sloter et al. 2000). A major advantage of the sperm ACM FISH assay over previous sperm FISH assays (Li et al. 2001; Liu S et al. 2000; Liu XX et al. 2003; Zhao et al. 2004) is that in addition to partial chromosomal duplications and deletions, which are induced in spermatogonia or meiosis, it detects chromosomal breaks induced during postmeiosis.

## Methods

*Study population and design.* Details of the study design, recruitment, and exposure assessment have been previously reported (Xing et al. 2010). All men who participated in the China Benzene and Sperm Study (C-BASS) were recruited from factories in Tianjin, China. Briefly, exposed men were recruited from three factories that used benzene-containing glues to manufacture shoes, paper bags, and sandpaper. Unexposed men were recruited from a meat-packing plant and an ice cream manufacturing factory, both of which had no history of benzene use. Eligibility criteria included age of 18–50 years, employment at the factory for at least 1 year, and no history of cancer or vasectomy. Informed consent was obtained from eligible men before enrollment in the exposure assessment phase of the study. All study materials were approved by the Committees for the Protection of Human Subjects at the University of California–Berkeley, Lawrence Livermore National Laboratory, Lawrence Berkeley National Laboratory, and the Tianjin Occupational Disease Hospital (Tianjin Third Municipal Hospital) under an institutional review board authorization agreement with the National Institute of Occupational Health and Poison Control, Chinese Center for Disease Control and Prevention. Questionnaires and other study materials were developed in English, translated to Mandarin, and reverse-translated to English.

Exposure assessment consisted of personal passive air badge monitoring (3M Organic Vapor Monitor, Model 3500; 3M, St. Paul, MN, USA) and spot urine samples. Both sample types were collected on two different occasions approximately 1 month apart. We measured benzene in air samples (parts per million) and urine samples (micrograms per liter) for both exposure groups. In addition, we measured *trans,trans*-muconic acid (MA) in the urine samples from exposed men. Details of benzene measurements in air and urine samples were published elsewhere (Xing et al. 2010). The limits of detection (LODs) were 0.2 ppm for air benzene, 0.016 µg/L for urinary benzene, and 10 mg/L for urinary MA.

*Sperm ACM-FISH analysis.* Semen samples were collected an average ± SD of 3.7 ± 2.2 days after the second urine collection from 78 men (34 exposed, 44 unexposed) of the 96 men originally enrolled in the study. Semen samples were aliquoted and stored at –80ºC without preservative. Sperm ACM-FISH was performed on samples from a subset of men: 10 men from each exposed group (low, moderate, and high) and 11 men from the unexposed group. At the time men were selected for the analysis, the urinary MA data were not yet available. Therefore, the 30 exposed men were selected based on their measured concentrations of urinary benzene such that 10 men were from the lowest tertile of urinary benzene concentration (low), 10 from the middle tertile (moderate), and 10 from the highest tertile (high). These three groups of men were then frequency-matched on age and smoking status in the 3 months before semen collection (yes/no), with unexposed men such that age distribution and smoking habits were balanced in each of the four groups.

The sperm ACM-FISH assay detects both structural (e.g., breaks, duplications, and deletions of the telomeric and centromeric regions of chromosome 1) and numerical abnormalities, using probes for three repetitive sequence regions of chromosome 1: 1cen (D1Z5, alpha, or A), 1q12 (pUC1.77, classical, or C), and 1p36.3 (D1Z2, midi, or M). Semen samples were thawed to room temperature and gently mixed with a Pasteur pipette, and a 5-mL aliquot was smeared onto a glass microscope slide and allowed to air-dry for 2 days. The sperm decondensation and hybridization procedures were as previously described (Sloter et al. 2000), except that the 1cen alpha satellite probe was derived from a purified plasmid clone, pSD21-1 (Waye et al. 1987) and directly labeled with tetramethylrhodamine-6-dUTP (Roche Applied Science, Indianapolis, IN, USA) using the GIBCO BRL Nick Translation System (Life Technologies, Inc., Gaithersburg, MD, USA). Slides were scored on a Zeiss Axioplan fluorescence microscope, using a 100× Plan-NEOFLUAR Ph3 objective (Carl Zeiss Microscopy, LLC, Thornwood, NY, USA), and equipped as previously described (van Hummelen et al. 1996).

Samples from 41 men (30 exposed, 11 unexposed) were analyzed by a single expert scorer who was blind to exposure status, using the sperm ACM-FISH assay and strict scoring criteria (Sloter et al. 2000). Slides were deidentified and coded by a technician other than the scorer. Five thousand sperm were then scored from the top half of the slide. Slides were recoded, and another 5,000 sperm were scored from the bottom half of the slide, for a total of approximately 10,000 sperm per slide. For quality control, the two sets of scores per participant for the aggregate types of anomalies, such as total structural anomalies and total breaks, were compared using Cochran’s equal-proportions test and data were accepted only if the *p*-value was > 0.05. In this study, no slide failed to meet this criterion.

*Statistical methods.* Demographic characteristics for the unexposed and exposed men were tabulated, and differences between the groups were examined using chi-square or Fisher’s exact tests (exact test was used if categories contained < 5 observations). The correlations were highly positive between the benzene levels measured on the two personal passive air monitors and the benzene levels in the two urine samples (Spearman’s rho (*r*_S_) > 0.7, *p* < 0.0005 for each pair); hence, the geometric mean (GM) of the two measurements was used as the summary value for each measure of exposure (air, urinary benzene, and urinary MA) for each participant. We then derived the GM, geometric standard deviation (GSD), and the percentiles of the distribution for each measure of exposure among participants classified as unexposed, exposed, and low, moderate, or high exposed. All 11 unexposed men and 1 low-exposed man had air benzene concentrations < LOD. The air benzene concentration for the low-exposed man was imputed as LOD/_√_^–^2 to calculate the GM and GSD for air benzene among men in the low-exposed group (Hornung and Reed 1990).

The dependent variables from the sperm ACM-FISH assay include duplications of 1p36.3, deletions of 1p36.3, duplications of the 1cen region, deletions of the 1cen region, breaks in 1q12, breaks between 1q12 and 1cen, and numerical aberrations including diploidy/disomy of chromosome 1 and chromosome 1 nullisomy. Because only one chromosome is examined in this assay, diploidy cannot be distinguished from disomy of chromosome 1. Counts of some anomalies were combined to summarize total duplications (1p36.3 + 1cen), deletions (1p36.3 + 1cen), breaks (breaks in 1q12 + breaks between 1q12 and 1cen), structural aberrations (all duplications, deletions, and breaks), and numerical aberrations (diploidy/disomy + nullisomy). The detection frequency of each anomaly (percentage of men with at least one anomaly per 10,000 sperm for each outcome) and the overall median, mean, and range of each anomaly/10,000 sperm were determined for each exposure group.

Separate standard negative binomial models were used to estimate incidence rate ratios (IRRs) and 95% confidence intervals (CIs) for the frequency of each ACM outcome (counts/10,000 sperm) among low-, moderate-, and high-exposed men compared with unexposed men. Zero-inflated negative binomial models were not deemed appropriate because of the small sample sizes even though some ACM outcomes had a moderate to large number of zero counts. We considered as potential confounders variables that were associated with benzene exposure, semen quality, or genetic damage in the literature [see Supplemental Material, Table 1 (http://dx.doi.org/10.1289/ehp.1103921)]. These included age (continuous), abstinence (continuous and ≤ 5 days vs. > 5 days), body mass index (< 18.5, 18.5–24.9, and 25–29.9 kg/m^2^), history of chronic disease (including self-reported history of high blood pressure or other diseases of the heart or blood vessels; tuberculosis; lung disease; anemia or other blood diseases; diabetes, thyroid diseases, or other hormonal diseases; stomach ulcers or other diseases of the GI tract; hepatitis or liver disease; epilepsy or other neurological disorders; and other chronic diseases), education (middle school or less vs. high school or more, use of hot baths in the 3 months before semen collection (yes/no), hours spent biking (more vs. less than 0.5 hr/day), and consumption of tea, cola, multivitamins, alcohol (yes/no in the 3 months before semen collection), and fruits and vegetables per day (more or less than the median of 3.6 times eaten per day). We included covariates in models if they were associated with exposure and outcomes (at *p* < 0.2) using separate bivariate negative binomial models or if the coefficients changed by more than 10% upon removal of the covariate. Although the exposure categories were frequency-matched on age and smoking in the 3 months before semen collection (yes/no), these two variables were included in all models to control for any residual confounding. For simplicity, the set of covariates that was associated with > 50% of the ACM outcomes (age, smoking, alcohol consumption, and history of chronic disease) was applied to all models. All others were excluded from models after determining whether inclusion changed coefficients by > 10%. We did not adjust models for education because this variable was collinear with exposure such that men with lower education had higher exposure. Abstinence was not associated with exposure and was associated with only one ACM outcome. Inclusion of abstinence as a covariate did not change coefficients; therefore, it was not retained in models. Associations with *p*-values < 0.05 were considered statistically significant.

**Table 1:** Distributions of benzene exposure measurements (air, urinary benzene, and urinary MA) among benzene-exposed and -unexposed workers.*^a^*

Percentiles								
Exposure measurement	*n*	GM (GSD)		Minimum	10th	25th	50th	75th	90th	Maximum
Urinary benzene (µg/L)																			
Unexposed		11		0.1	(1.3)		0.1		0.1		0.1		0.1		0.2		0.2		0.2
Exposed*b*		30		14.9	(5.0)		0.8		2.4		4.3		11.0		59.0		120.5		617.0
Low exposed		10		2.8	(1.7)		0.8		1.4		2.4		2.9		4.3		5.0		5.4
Moderate exposed		10		11.6	(1.6)		6.7		7.0		7.2		11.0		21.0		21.3		21.3
High exposed		10		102.4	(2.1)		48.5		49.2		59.0		110.6		123.1		373.9		617.0
Total		41		4.1	(12.8)		0.1		0.1		0.2		5.4		21.3		114.9		617.0
Air (ppm)																			
Unexposed		11		—			< LOD		< LOD		< LOD		< LOD		< LOD		< LOD		< LOD
Exposed*b,c*		30		2.8	(3.6)		< LOD		0.7		1.0		3.3		7.0		16.3		23.6
Low exposed*c*		10		1.0	(2.6)		< LOD		0.3		0.7		1.2		2.1		3.1		4.1
Moderate exposed		10		3.0	(3.4)		0.6		0.7		0.8		3.7		7.0		17.2		23.6
High exposed		10		7.6	(2.2)		2.0		2.8		4.4		8.4		14.1		19.4		20.2
Total*c*		41		1.3	(5.6)		< LOD		< LOD		< LOD		1.3		4.7		11.6		23.6
Muconic acid (mg/L)																			
Unexposed		0		—			—		—		—		—		—		—		—
Exposed*b*		30		5.3	(3.3)		0.8		1.2		1.9		6.6		13.8		25.2		40.9
Low exposed		10		1.7	(1.6)		0.8		0.9		1.2		1.7		2.7		3.2		3.3
Moderate exposed		10		5.4	(3.1)		1.1		1.3		1.9		7.9		14.4		19.1		23.7
High exposed		10		15.6	(1.8)		6.5		7.6		11.2		13.4		26.6		34.0		40.9
Total		30		5.3	(3.3)		0.8		1.2		1.9		6.6		13.8		25.2		40.9
—, not measured. **a**Number of men (*n*), GM, GSD, percentiles, and range of concentrations (minimum, maximum) among men. Urine samples and personal air measurements were obtained from each man at two time points approximately 1 month apart, and the GMs of the paired measurements were used to calculate summary statistics. **b**Exposure categories are based on tertiles of urinary benzene concentrations among exposed men (low: 0.8–5.4, moderate: 6.7–21.3, high: 48.5–617.0 µg/L). **c**To estimate the GM and GSD, values < LOD (all 11 unexposed men and 1 low-exposed man) were imputed as LOD/√–2.																			

Finally, we used negative binomial models to perform a trend analysis (separately for each outcome) where the urinary benzene explanatory variable was coded as 0 for unexposed, 1 for low exposed, 2 for moderate exposed, and 3 for high exposed. Trend analysis models were adjusted by age, smoking, alcohol consumption, and history of chronic disease and are reported as *p*_trend_.

## Results

The population characteristics of the exposed and unexposed men analyzed in the present study [see Supplemental Material, Table 1 (http://dx.doi.org/10.1289/ehp.1103921)] were similar with the exception of education, such that unexposed men were more likely to have completed high school than exposed men (55% compared with 10%, *p* = 0.006). Overall, men smoked an average of 10 ± 9 cigarettes/day in the 3 months before semen collection; because men were frequency matched on whether they smoked in the 3 months before semen collection, this variable did not differ by exposure group (73% and 77% of unexposed and exposed men smoked within the previous 3 months, respectively). Duration of abstinence was similar in the two groups (mean ± SD: 6.4 ± 5.4 vs. 6.6 ± 3.7 days, unexposed and exposed, respectively). Two unexposed men reported a doctor-diagnosed fertility problem, yet one had fathered a child. Semen quality and the percentages of men who had below-normal values for sperm concentration, sperm count, semen volume, and percent sperm motility according to World Health Organization (WHO) criteria (WHO 1992) did not differ significantly by exposure group (see Supplemental Material, Table 2).

**Table 2:** Percentage of men with at least one abnormality detected as determined by sperm ACM-FISH of chromosome 1, and median (p50), mean, range of anomaly frequencies (counts/10,000 sperm),*^a^* stratified by benzene exposure group.

Unexposed (*n* = 11)					Low exposed (*n* = 10)					Moderate exposed (*n* = 10)					High exposed (*n* = 10)				
Outcome	With anomaly (%)	p50	Mean (range)		With anomaly (%)	p50	Mean (range)		With anomaly (%)	p50	Mean (range)		With anomaly (%)	p50	Mean (range)	
Total anomalies		100	22.9		22.6	(13.0–42.6)		100	27.9		28.2	(16.9–50.3)		100	29.9		30.0	(17.9–48.8)		100	33.2		34.8	(15.9–48.8)
Total structural aberrations		100	12.9		12.8	(6.9–19.0)		100	15.4		17.1	(11.0–26.6)		100	19.4		17.9	(11.0–23.9)		100	21.4		21.2	(11.9–32.9)
Total 1p36.3 & 1cen dup/del		73	2.0		2.3	(0.0–7.9)		100	4.5		4.9	(1.0–10.0)		100	6.0		5.4	(2.0–10.9)		100	6.0		5.9	(3.0–9.0)
Total dup		64	1.0		1.9	(0.0–6.9)		100	2.5		3.7	(1.0–10.0)		100	3.5		3.9	(1.0–8.9)		100	4.0		3.9	(1.0–6.0)
Total del		36	0.0		0.4	(0.0–2.0)		60	1.0		1.2	(0.0–4.0)		80	1.5		1.5	(0.0–3.0)		100	1.0		2.0	(1.0–5.0)
Total 1p36.3 dup/del		64	1.0		1.3	(0.0–5.0)		100	2.5		3.4	(1.0–9.0)		90	3.5		3.3	(0.0–5.0)		100	4.0		4.3	(2.0–8.0)
1p36.3 dup		64	1.0		1.0	(0.0–4.0)		70	1.5		2.2	(0.0–6.0)		80	2.0		1.9	(0.0–4.0)		100	2.5		2.6	(1.0–6.0)
1p36.3 del		27	0.0		0.3	(0.0–1.0)		60	1.0		1.2	(0.0–4.0)		80	1.5		1.4	(0.0–3.0)		80	1.0		1.7	(0.0–4.0)
Total 1cen dup/del		55	1.0		1.1	(0.0–3.0)		60	1.5		1.5	(0.0–4.0)		80	1.5		2.1	(0.0–6.9)		90	1.0		1.6	(0.0–4.0)
1cen dup		45	0.0		0.9	(0.0–3.0)		60	1.5		1.5	(0.0–4.0)		70	1.5		2.0	(0.0–6.9)		60	1.0		1.3	(0.0–4.0)
1cen del		9	0.0		0.2	(0.0–2.0)		0	0.0		0.0	(0.0–0.0)		10	0.0		0.1	(0.0–1.0)		30	0.0		0.3	(0.0–1.0)
Total breaks		100	8.9		10.5	(5.8–18.0)		100	12.4		12.2	(8.0–19.7)		100	12.9		12.5	(7.0–17.9)		100	14.4		15.3	(8.9–23.9)
1q12*b*		100	3.0		4.0	(1.0–13.0)		70	3.5		4.1	(0.0–11.0)		100	4.5		4.9	(1.0–9.0)		100	7.9		7.7	(3.0–12.9)
1cen-1q12*c*		100	5.0		6.5	(3.9–14.0)		100	8.9		8.2	(3.0–11.0)		100	7.9		7.7	(3.0–11.0)		100	6.5		7.6	(3.9–13.8)
Total numerical aberrations		100	8.0		9.5	(1.0–27.7)		100	9.5		10.7	(2.0–22.7)		100	10.5		11.6	(5.0–25.9)		100	13.8		12.7	(3.0–20.9)
Disomy 1		100	8.0		9.4	(1.0–27.7)		100	9.0		10.6	(2.0–22.7)		100	10.5		11.6	(5.0–25.9)		100	13.4		12.6	(3.0–20.9)
Nullisomy 1		9	0.0		0.1	(0.0–1.0)		10	0.0		0.1	(0.0–1.0)		0	0.0		0.0	(0.0–0.0)		10	0.0		0.1	(0.0–1.0)
Other*d*		18	0.0		0.3	(0.0–2.0)		40	0.0		0.4	(0.0–1.0)		40	0.0		0.4	(0.0–1.0)		50	0.5		0.9	(0.0–4.0)
Abbreviations: del, deletions; dup, duplications; p50, median. **a**Sperm structural and numerical defects were determined using the sperm ACM-FISH assay of chromosome 1. Frequencies per 10,000 sperm counted are reported. Percentage includes men with at least one sperm with this defect per 10,000 sperm analyzed. Median and mean frequencies include all participants, and men without a detected anomaly were assigned a value of zero. **b**Breaks within 1q12. **c**Breaks between 1cen and 1q12. **d**“Other” is defined as all anomalies not detailed above.																								

All three measures of exposure (air benzene, urinary benzene, and urinary MA) were highly correlated among exposed men (*r*_S_ > 0.8, *p* < 0.0005 for each pair). Median urinary benzene measurements were 2.9, 11.0, and 110.6 µg/L, respectively, for men in the low-, moderate-, and high-exposed groups compared with 0.1 µg/L for unexposed men (Table 1). Median air benzene concentrations were 1.2, 3.7, and 8.4 ppm among the low-, moderate-, and high-exposed groups, respectively, compared with < LOD for all unexposed men. Five men in the low-exposed group and three men in the moderate-exposed group had air benzene measurements below the U.S. PEL of 1 ppm. Finally, median concentrations of MA were 1.7, 7.9, and 13.4 mg/L for low-, moderate-, and high-exposed men, respectively. MA was not measured in unexposed men.

Structural aberrations (1p36.3 and 1cen duplications and deletions, breaks in 1q12, and breaks between 1p12 and 1cen) and numerical aberrations (diploidy/disomy or nullisomy) of chromosome 1 were found in sperm of all men participating in the study, and aberration counts/10,000 sperm increased with exposure category (Table 2). Two ACM outcomes, 1cen deletions and nullisomy, were rare and were excluded from further analyses.

Occupational exposure to benzene was associated with chromosomal structural aberrations, but not numerical abnormalities (Table 3). Adjusted IRRs for all structural aberrations combined were 1.42 (95% CI: 1.10, 1.83), 1.44 (95% CI: 1.12, 1.85), and 1.75 (95% CI: 1.36, 2.24) in the low-, moderate-, and high-exposed men compared with unexposed men. Among the structural aberrations, associations increased with increasing categorical benzene exposure for combined 1p36.3 duplications and deletions (*p*_trend_ < 0.005), but not the 1cen region (*p*_trend_ = 0.19). Compared with unexposed men, adjusted IRRs for 1p36.3 duplication were 2.31 (95% CI: 1.03, 5.18), 2.13 (95% CI: 0.95, 4.79), and 3.12 (95% CI: 1.39, 6.97) for the low-, moderate-, and high-exposed men, respectively. Associations were stronger for 1p36.3 deletion, with adjusted IRRs of 4.31 (95% CI: 1.18, 15.78), 6.02 (95% CI: 1.69, 21.39), and 7.88 (95% CI: 2.21, 28.05) for the low-, moderate-, and high-exposed groups compared with unexposed men. Results were similar when benzene exposure was categorized according to tertiles of urinary MA [see Supplemental Material, Table 3 (http://dx.doi.org/10.1289/ehp.1103921)].

**Table 3:** Adjusted*^a^* associations between urinary benzene exposure*^b^* and sperm ACM-FISH outcomes.

Low vs. unexposed				Moderate vs. unexposed				High vs. unexposed			
Outcome	IRR (95% CI)		*p-*Value	IRR (95% CI)		*p-*Value	IRR (95% CI)		*p-*Value	*p*_trend_*c*
Total anomalies		1.33	(1.00, 1.75)		0.05		1.38	(1.05, 1.82)		0.02		1.69	(1.28, 2.23)		< 0.005		< 0.005
Total structural aberrations		1.42	(1.10, 1.83)		0.01		1.44	(1.12, 1.85)		0.01		1.75	(1.36, 2.24)		< 0.005		< 0.005
Total 1p36.3 & 1cen dup/del		2.22	(1.35, 3.66)		< 0.005		2.53	(1.56, 4.11)		< 0.005		2.96	(1.81, 4.85)		< 0.005		< 0.005
Total dup		2.19	(1.15, 4.17)		0.02		2.31	(1.23, 4.33)		0.01		2.51	(1.31, 4.82)		0.01		0.01
Total del		2.66	(0.91, 7.76)		0.07		3.68	(1.31, 10.33)		0.01		5.25	(1.90, 14.55)		< 0.005		< 0.005
Total 1p36.3 dup/del		2.72	(1.43, 5.19)		< 0.005		2.92	(1.54, 5.54)		< 0.005		4.07	(2.16, 7.66)		< 0.005		< 0.005
1p36.3 dup		2.31	(1.03, 5.18)		0.04		2.13	(0.95, 4.79)		0.07		3.12	(1.39, 6.97)		0.01		0.01
1p36.3 del		4.31	(1.18, 15.78)		0.03		6.02	(1.69, 21.39)		0.001		7.88	(2.21, 28.05)		< 0.005		< 0.005
Total 1cen dup/del		1.67	(0.70, 4.01)		0.25		2.07	(0.91, 4.73)		0.08		1.75	(0.71, 4.27)		0.22		0.19
1cen dup		2.00	(0.69, 5.82)		0.20		2.47	(0.87, 6.97)		0.09		1.75	(0.57, 5.44)		0.33		0.28
1cen del*d*		—			—		—			—		—			—		—
Total breaks		1.24	(0.91, 1.69)		0.18		1.20	(0.89, 1.63)		0.24		1.49	(1.10, 2.02)		0.01		0.02
1q12*e*		1.29	(0.68, 2.45)		0.44		1.27	(0.69, 2.34)		0.44		2.23	(1.22, 4.09)		0.01		0.01
1cen-1q12*f*		1.22	(0.86, 1.74)		0.26		1.17	(0.83, 1.67)		0.37		1.14	(0.79, 1.63)		0.49		0.56
Total numerical aberrations		1.21	(0.71, 2.07)		0.49		1.32	(0.78, 2.25)		0.30		1.59	(0.92, 2.76)		0.10		0.10
Disomy 1		1.22	(0.71, 2.10)		0.47		1.35	(0.79, 2.30)		0.28		1.61	(0.92, 2.81)		0.10		0.10
Nullisomy 1*d*		—			—		—					—			—		—
Other*g*		1.40	(0.28, 6.92)		0.68		1.34	(0.28, 6.47)		0.72		2.95	(0.71, 12.30)		0.14		0.13
**a**Multivariable negative binomial models were used to estimate IRRs. IRRs represent comparisons of counts/10,000 sperm. All models were adjusted for age, smoking, or alcohol consumption in the 3 months before semen collection and for history of any chronic disease. **b**Urinary benzene concentrations (summarized by the GM of the two measurements) among the exposed were divided into three groups with 10 men in each. Statistical models compared each exposure group with the unexposed group. **c***p*_trend_ is the *p*-value given by an adjusted negative binomial regression model where the urinary benzene explanatory variable was coded as 0 for unexposed, 1 for low exposed, 2 for moderate exposed, and 3 for high-exposed. **d**Models were not constructed because of low detection frequency. **e**Breaks within 1q12. **f**Breaks between 1cen and 1q12. **g**“Other” is defined as all anomalies not detailed above.																	

Benzene exposure was also associated with chromosomal breaks, but the association with all breaks combined was significant for the high-exposure benzene group only [IRR 1.49 (95% CI: 1.10, 2.02)] (Table 3). This association appeared to be specific for breaks within the 1q12 region [IRR 2.23 (95% CI: 1.22, 4.09) for high exposure compared with no exposure); there was no clear association with breaks between the 1cen and 1q12 region.

An analysis comparing the eight men exposed to < 1 ppm of air benzene, the current U.S. PEL, to the 11 unexposed men indicated significant associations with 1p36.3 duplications and deletions [IRR 3.36 (95% CI: 1.66, 6.81)], 1cen duplications and deletions [3.38 (95% CI: 1.62, 7.09)], and all structural aberrations (duplications, deletions, and breaks) combined [1.53 (95% CI: 1.17, 1.99)]. However, total breaks were not associated with exposure (data not shown).

No significant associations between benzene exposure and numerical abnormalities were observed (Table 3).

## Discussion

We previously reported evidence that occupational exposure to benzene, even at levels near the OSHA PEL of 1 ppm, was associated with aneuploidy for chromosomes X, Y, and 21 in sperm (Xing et al. 2010). Here, in a subsample of the same study population, we report that occupational exposure to benzene is also positively associated with structural aberrations of chromosome 1 in sperm. Even men occupationally exposed to benzene in air at levels < 1 ppm had higher frequencies of sperm with structural chromosomal aberrations than unexposed controls. We did not find associations with aneuploidy of chromosome 1; however, the sperm ACM-FISH assay does not effectively distinguish between disomy 1 and diploidy (Sloter et al. 2000). Therefore, this result should not be interpreted to mean that benzene does not induce aneuploidy for chromosome 1 in sperm.

Our finding of increasing associations in sperm with duplications and deletions of chromosome 1 with increasing exposure is consistent with previous findings in men who were exposed to higher levels of benzene (mean 10.81 ppm vs. 5.57 ppm among all exposed men in our study) (Liu et al. 2003). More important, our study suggests that benzene may induce chromosomal damage in sperm at low doses. Because the ability to detect this kind of damage in sperm by FISH is unique to the sperm ACM-FISH assay, no previous data are available for comparison; however, men exposed to high concentrations of benzene (mean = 22.12 ppm) were found to have increased DNA breaks in sperm using the single-cell gel electrophoresis assay (Song et al. 2005). The clastogenic effects of benzene are thought to result from a combination of DNA adduct formation, oxidative damage, and inhibition of topoisomerase II activity (Whysner et al. 2004).

Duplications and deletions of 1q36.3 and 1q12 detected by our assay occur before the two meiotic segregation divisions. Two plausible mechanisms of origin are possible. First, benzene may induce breaks during meiosis, resulting in random segregation of the broken fragments during meiotic chromosome segregation. Second, benzene may generate DNA damage in stem cells/spermatogonia that are misrepaired into balanced chromosomal rearrangements (reciprocal translocations or inversions involving 1p). In the first case, duplications and deletions represent events induced during an approximately 25-day window, the duration of meiosis in humans (Adler 1996), which occurs between approximately 40 and 65 days before semen collection. In the second case, chromosomal rearrangements would form quadrivalents during meiosis I and generate unbalanced gametes carrying partial chromosomal duplications or deletions (van Hummelen et al. 1997). Sperm carrying duplications and deletions generated by this mechanism would persist for as long as the stem cell carrying the balanced rearrangement survives in the seminiferous epithelium, which could be the entire reproductive lifetime of the individual. This mechanism thus represents persistent benzene-induced sperm damage that can extend far beyond the end of the exposure window. Further research will be needed to discriminate between duplications and deletions generated through breaks induced in meiosis or through chromosomal rearrangements in stem cells/spermatogonia. Studies of men who are no longer exposed to benzene but have a history of occupational exposure to benzene could help distinguish between these two mechanisms.

Chromosomal breaks detected by the sperm ACM-FISH assay likely originated during the postmeiotic phase of spermatogenesis, a window of approximately 5 weeks before semen collection (Adler 1996). This is a period known to be sensitive for the induction of DNA damage and chromosomal breaks (Marchetti and Wyrobek 2008). However, we hypothesize from our results that occupational exposure to benzene must reach a threshold before a noticeable number of breaks are induced and that exposure to levels of benzene near the U.S. PEL is not likely to induce elevated numbers of breaks during this time window. This finding supports the conclusion that the majority of duplications and deletions observed in the low-exposure groups are most likely the result of accumulated chromosomal rearrangements in stem cells/spermatogonia rather than breaks induced during the meiotic cell cycle that produced the sampled sperm. Therefore, the effects of occupational exposure to benzene may not be reversible because stem cells with chromosomal rearrangements would continue to produce a lifetime of unbalanced gametes at every cycle of spermatogenesis, even after the end of occupational exposure to benzene (i.e., a worker leaves that job).

The sperm ACM-FISH assay provides a direct assessment of chromosomal damage for the portion of the genome contained within the 1p36 to 1q12 regions, which represents 4–5% of the haploid genome (Mendelsohn et al. 1973). Regardless of exposure status, duplications and deletions of 1p36.3 were more common than duplications and deletions of 1cen and, as a whole, duplications and deletions were 4–5 times less common than breaks. In addition, the frequencies of sperm with duplications were slightly higher than the frequencies of sperm with deletions for the same chromosomal region. These findings are in agreement with previous studies of healthy men (Bosch et al. 2003; Sloter et al. 2000, 2004) and support the accuracy of the scoring criteria used in this study.

Our study identifies occupational exposure to benzene as a possible risk factor in the etiology of the 1p36 deletion syndrome. Monosomy of 1p36 (terminal deletion of chromosome 1) is an established cause of mental retardation and major congenital malformations in children, with an incidence of 1–2 children per 10,000 (Shaffer and Heilstedt 2001; Zenker et al. 2002). In our study, adjusted IRRs for 1p36.3 deletions were 4.31, 6.02, and 7.88 for the low-, moderate-, and high-exposed groups, respectively. The relationship between benzene exposure and 1p36 deletion appears to be stronger than the association between sperm aneuploidy and benzene exposure (where adjusted IRRs for disomy X, the strongest association observed, were 2.0 and 2.8 in low- and high-exposed men, respectively, vs. unexposed men) observed in the same population (Xing et al. 2010). This supports the notion that the clastogenic activity of benzene is stronger than its aneugenic activity (Chen et al. 1994).

The sperm ACM-FISH assay provides an indirect assessment of the damage in the rest of the genome on the assumption that the area targeted by this assay is, on average, representative of the rest of the genome. This assumption is supported by studies that have found good concordance between the frequencies of sperm with chromosomal aberrations as detected by the human sperm/hamster-egg technique, which detects aberrations in all sperm chromosomes (Rudak et al. 1978), and the frequencies obtained by extrapolating to the whole genome the frequencies of sperm with structural aberrations as detected by the sperm ACM-FISH assay (Sloter et al. 2000). Extrapolating the ACM data to the whole genome yields estimated frequencies of sperm with chromosomal structural aberrations of 2.6–3.2, 3.4–4.3, 3.6–4.5, and 4.3–5.3% for unexposed, low-, moderate-, and high-exposed groups, respectively.

A limitation of our study is that occupational exposure to benzene was monitored only twice during an approximate 1-month period before semen sample collection. Because some of the damage measured by our assay may have occurred at an earlier time point than that monitored by our exposure assessment, it remains possible that exposure may have been misclassified. However, we consider this to be of minimal impact. To limit misclassification of exposure, participants were required to have worked at the same factory for at least a year to be enrolled in the study. In addition, we found a very strong correlation among all measurements of exposure (air benzene, urinary benzene, and urinary MA) and between the two times of exposure assessment (Xing et al. 2010), suggesting that the measured exposure levels represented the usual workplace exposure level for each man. Because of the nature of the sperm ACM-FISH assay, we performed many tests of association for all of the different outcomes and groups of outcomes. Thus, it is possible that some of our findings may be due to random chance. A final limitation is that recruitment from an occupational setting may predispose our population to biases such as the “healthy worker survival effect,” whereby exposed men who were eligible for recruitment may have relatively low susceptibility to the effects of benzene (Garcia and Checkoway 2003). This bias may have underestimated associations. In addition, uncontrolled confounding is possible because of the inherent differences between the exposed and unexposed populations.

## Conclusions

We found that that occupational exposure to benzene, even at levels at or below the U.S. PEL, was associated with chromosomal abnormalities in sperm that have been associated with infertility and spontaneous abortions and with mental retardation and inherited defects in children. Our findings suggest that benzene may be a possible risk factor for *de novo* 1p36 deletion syndrome and raise the possibility that occupational exposure to benzene may induce persistent chromosomal rearrangements in stem cells/spermatogonia that would continue to generate unbalanced gametes throughout the reproductive life of the exposed men, even after they are no longer exposed to benzene. These results, as well as our previous findings (Xing et al. 2010), indicate a need for more studies of the potential effects of low exposures (≤ 1 ppm) to benzene, especially chromosomal abnormalities in the germ line of exposed men, and a reevaluation of the current occupational exposure limit of 1 ppm by OSHA.

## Supplemental Material

(135 KB) PDF
