# Supplementary material for: Occupational Exposure to Benzene and Chromosomal Structural Aberrations in the Sperm of Chinese Men
Source: Environ Health Perspect. 2011 Nov 15;120(2):229–34. doi: 10.1289/ehp.1103921 (PMC3279447; doi:10.1289/ehp.1103921)
Supplement: (135 KB) PDF [file ehp.1103921.s001.pdf]

SUPPLEMENTAL MATERIAL

Occupational Exposure to Benzene and Chromosomal Structural Aberrations in the Sperm of Chinese Men

Francesco Marchetti, Brenda Eskenazi, Rosana H Weldon, Guilan Li, Luoping Zhang, Stephen M Rappaport, Thomas E Schmid, Caihong Xing, Elaine Kurtovich, and Andrew J Wyrobek

Supplemental Material Table 1. China Benzene and Sperm Study (C-BASS) population characteristics and associations[a, b] with exposure category.

	Unexposed		Exposed		
Characteristic	N	(%)	N	(%)	p-value
Age (years) [a]					
19-32	5	(45)	16	(53)	0.655
33-49	6	(55)	14	(47)	
Abstinence (days) [a]					
≤5	5	(45)	18	(60)	0.406
>5	6	(55)	12	(40)	
Body Mass Index (kg/m^2) [a]					
underweight (<18.5)	0	(0)	3	(10)	0.462
normal (18.5-24.9)	6	(55)	19	(63)	
overweight (25-29.9)	5	(45)	8	(27)	
Current tea drinker[b]					
No	10	(91)	22	(73)	0.401
Yes	1	(9)	8	(27)	
Current cola drinker[b]					
No	10	(91)	24	(80)	0.651
Yes	1	(9)	6	(20)	
Chronic disease[b, c]					
No	7	(64)	26	(87)	0.178
Yes	4	(36)	4	(13)	
Multivitamin use					
No	11	(100)	29	(97)	N/A
Yes	0	(0)	1	(3)	
Smoked in last 3 months[b]					
No	3	(27)	7	(23)	1.000
Yes	8	(73)	23	(77)	
Drank alcohol in last 3 months[b]					
No	1	(9)	6	(20)	0.651
Yes	10	(91)	24	(80)	
Taken hot baths in last 3 months[a]					
No	6	(55)	11	(37)	0.303
Yes	5	(45)	19	(63)	
Bikes 0.5 or more hours per day[a]					
No	5	(45)	10	(33)	0.475
Yes	6	(55)	20	(67)	
Eats fruit and vegetables > 3.6 times per day[b]					
No	7	(64)	14	(47)	0.484
Yes	4	(36)	16	(53)	
Education[b]					
Middle school or less	5	(45)	27	(90)	0.006
High school or more	6	(55)	3	(10)	

[a] χ^2 tests were used to assess differences in categorical variables between control and exposed groups. [b] Fisher's exact tests were used when categories contained fewer than 5 observations. [c] Chronic disease includes self-reported history of high blood pressure, other diseases of the heart or blood vessels, tuberculosis, lung disease, anemia, other blood diseases, diabetes, thyroid diseases, other hormonal diseases, stomach ulcers or other diseases of the GI tract, hepatitis, liver disease, epilepsy or other neurological disorders, or other chronic diseases.

Supplemental Material Table 2. Comparisons of descriptive statistics of semen quality parameters of men participating in the China-Benzene and Sperm Study .

	Unexposed (n=11)		Exposed (n=30)			Unexposed	Exposed	
Semen Quality parameter	Median	(p25, p75)	Median	(p25, p75)	p[b]	% <Normal[a]	% <Normal[a]	p[c]
Concentration (x 10^6/ml)	49.3	(35.3, 85.0)	53.9	(36.9, 88.6)	0.60	0	3	1
Count (x 10^6)	1133	(813, 1307)	1158	(801, 1884)	0.80	0	0	N/A[d]
Volume (ml)	2.7	(2.3, 3.6)	3.1	(2.1, 3.6)	0.96	18	20	1
Motility (%)	31	(21, 34)	38	(21, 46)	0.18	91	83	1

[a] Percent of men who were below the normal value according to the World Health Organization criteria. The WHO guidelines used were: concentration <$20x10^6$/ml, count<$40x10^6$, volume <2 ml, and motility <50% (W.H.O. 1992). [b] p-value given by a Wilcoxon rank sum test. [c] p-value given by a Fisher's exact test.
[d] No test could be performed since all men had normal sperm counts, but men did not differ by exposure group.

Supplemental Material Table 3. Adjusted[a] incidence rate ratios (IRR) for ACM outcomes and benzene exposure[b] according to tertiles of urinary muconic acid.

	Low vs. unexposed		Moderate vs. unexposed		High vs. unexposed		
Outcome	IRR (95% CI)	p	IRR (95% CI)	p	IRR (95% CI)	p	p_{trend}[f]
Total anomalies	1.48(1.12, 1.95)	0.01	1.25(0.94, 1.64)	0.12	1.68 (1.26, 2.23)	<0.005	<0.005
Total structural aberrations	1.49(1.16, 1.91)	<0.005	1.33(1.03, 1.70)	0.03	1.82 (1.42, 2.33)	<0.005	<0.005
Total 1p36.3 & 1cen dup/del	2.99(1.86, 4.82)	<0.005	1.76(1.05, 2.93)	0.03	3.05 (1.86, 5.03)	<0.005	<0.005
Total duplications	2.83(1.61, 5.00)	<0.005	1.50(0.81, 2.78)	0.20	2.63 (1.44, 4.83)	<0.005	0.04
Total deletions	3.71(1.32, 10.44)	0.01	2.95(1.02, 8.51)	0.05	4.99(1.75, 14.24)	<0.005	0.01
Total 1p36.3 dup/del	3.57(1.90, 6.68)	<0.005	2.15(1.10, 4.21)	0.03	4.05 (2.11, 7.77)	<0.005	<0.005
1p36.3 duplications	2.93(1.38, 6.25)	0.01	1.57(0.69, 3.57)	0.29	3.00 (1.36, 6.62)	0.01	0.05
1p36.3 deletions	5.99(1.69, 21.25)	0.01	4.37(1.18, 16.14)	0.03	8.04(2.21, 29.27)	<0.005	<0.005
Total 1cen dup/del	2.24(1.00, 5.04)	0.05	1.35(0.57, 3.20)	0.50	1.97 (0.82, 4.75)	0.13	0.27
1cen duplications	2.50(0.93, 6.75)	0.07	1.47(0.50, 4.33)	0.48	2.32 (0.75, 7.13)	0.14	0.29
1cen deletions[c]	-- --	--	-- --	--	-- --	--	--
Total breaks	1.16(0.84, 1.59)	0.37	1.23(0.91, 1.67)	0.18	1.55 (1.14, 2.11)	0.01	0.01
1q12[d]	1.23(0.64, 2.36)	0.54	1.26(0.68, 2.35)	0.46	2.28 (1.24, 4.20)	0.01	0.01
1cen-1q12[e]	1.13(0.78, 1.63)	0.52	1.23(0.86, 1.75)	0.26	1.18 (0.82, 1.71)	0.38	0.31
Total numerical aberrations	1.47(0.87, 2.50)	0.15	1.14(0.67, 1.97)	0.63	1.46 (0.82, 2.60)	0.19	0.33
disomy 1	1.48(0.87, 2.53)	0.15	1.15(0.67, 1.99)	0.61	1.49 (0.83, 2.66)	0.18	0.31
nullisomy 1[c]	-- --	--	-- --	--	-- --	--	--
Other	1.85(0.37, 9.29)	0.45	1.31(0.27, 6.50)	0.74	2.49(0.55, 11.24)	0.24	0.31

[a] Multivariable negative binomial models were used to estimate Incidence Rate Ratios (IRRs). IRRs represent comparisons of counts/10,000 sperm. All models were adjusted for age, smoking or alcohol consumption in the three months prior to semen collection and history of any chronic disease. [b] Muconic acid concentrations (summarized by the GM of the two measurements) among the exposed men with ACM analyses were divided into three equal groups of 10. Urinary muconic acid (in mg/L) ranged 0.8-2.1, 2.4-11.6, and 12.0-40.9 in low-, moderate-, and high-exposed men, respectively. Urinary muconic acid was not measured in unexposed men. Statistical models compared each exposure group with the unexposed group. [c]Models were not constructed due to low detection frequency. [d] breaks within 1q12; [e] breaks between 1cen and 1q12; [f] p_{trend} is the p-value given by an adjusted negative binomial regression model where the urinary muconic acid explanatory variable was coded as 0 for unexposed, 1 for low-exposed, 2 for moderate-exposed and 3 for high-exposed.
